# Supplementary material for: Computational identification of cell-specific variable regions in ChIP-seq data
Source: Nucleic Acids Res. 2020 Mar 18;48(9):e53. doi: 10.1093/nar/gkaa180 (PMC7229859; doi:10.1093/nar/gkaa180)

# Supplementary Material

# Conversion of JSON-based files to a relational SQL database

The metadata in ENCODE is represented in JSON-format. There is an API (Application Program Interface) that enables downloading JSON files for many experiments automatically. In addition, it is possible to restrict the experiments to be downloaded by specifying conditions the experimental metadata has to comply with. However, a JSON file for one experiment can include up to 11,000 lines and this can cause two problems. The first is that the extraction of information can be complicated and requires further implementation of small scripts / programs. The second is that parsing such files can be computationally expensive especially if there are more than 1000 files to be processed. To avoid these problems and enabling the analysis of the metadata in a flexible and fast way we decided to convert the information from JSON into a MySQL database. For objects like experiment, target, organism, replicate, etc. we downloaded all JSON files. A Python script was implemented that extracts information encoded in each JSON file and automatically fills the SQL database with this information. The procedure in the Python script takes also cares about creating the tables for each object and furthermore creating also relational tables for the connection between two tables (objects). Relational information is also automatically extracted and stored into SQL. Figure1 represents a simplified case with one experiment. The brackets ”{}” define the beginning and end of a JSON. There are simple features like accession and status for which the value is directly given. Those simple values can also appear in lists represented by the brackets ”[]”. However, one JSON can include other JSONs. This is shown by the target in the experiment that is completely described by a so called nested JSON. It is also possible that one JSON includes a list of JSONs. The example in Figure S1 shows that for each file that is related to the experiment there is one JSON within the experiment. These nested JSONs are the reason why those files are getting so large. Another advantage coming with the conversion from JSON into SQL is that objects are not stored redundantly. Taking as example the target that describes the protein that is targeted in a ChIP-seq experiment. All JSONs for experiments with the same target store the same information for the same target multiple times. In SQL there is only one entry for a specific target in the target table and experiments with this target are just linked to this entry by the target_ID. An example for such a link is represented by the target in Supplementary Figure S1 below and has_file is a relational table.

**Figure S1**


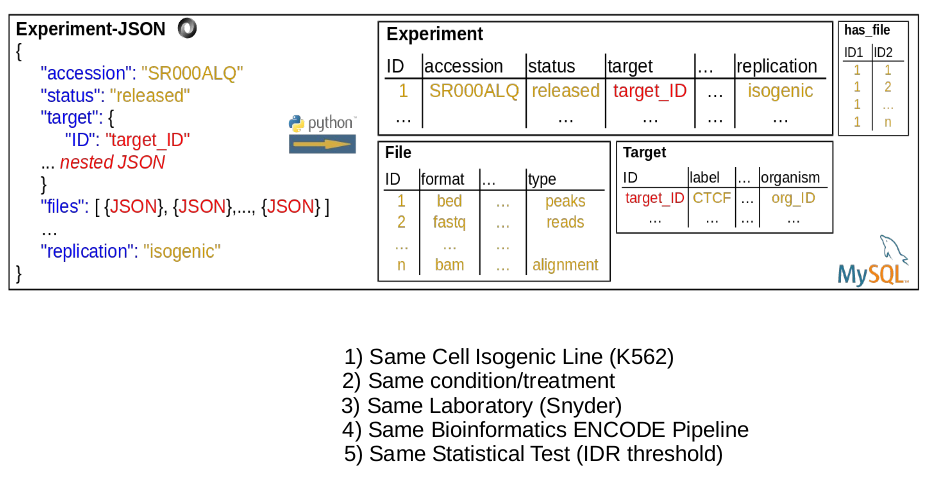


# Figure S2

# (A) DNA binding motifs of the protein targets of the ChIP-seq samples used to detect variable regions in mouse ESCs from the Jaspar database. No data for ZC3H11A and HCFC1.

#
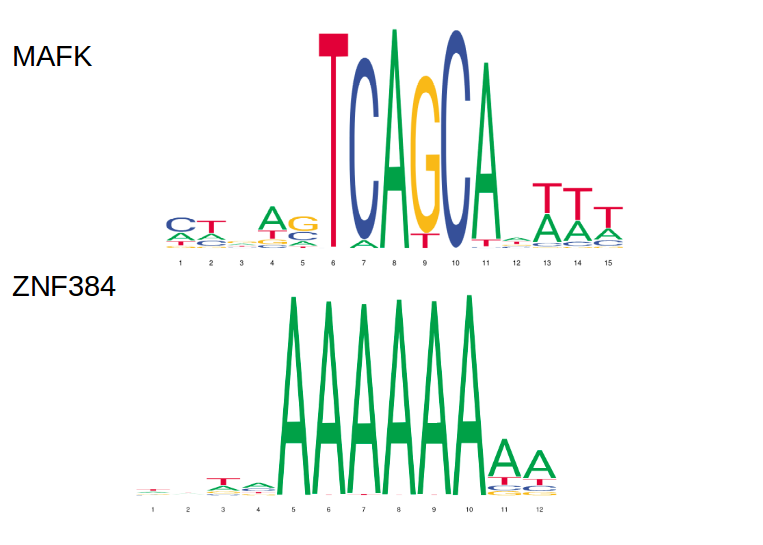


# (B) DNA binding motifs of the protein targets of the ChIP-seq samples used to detect variable regions in K562 cell lines

#
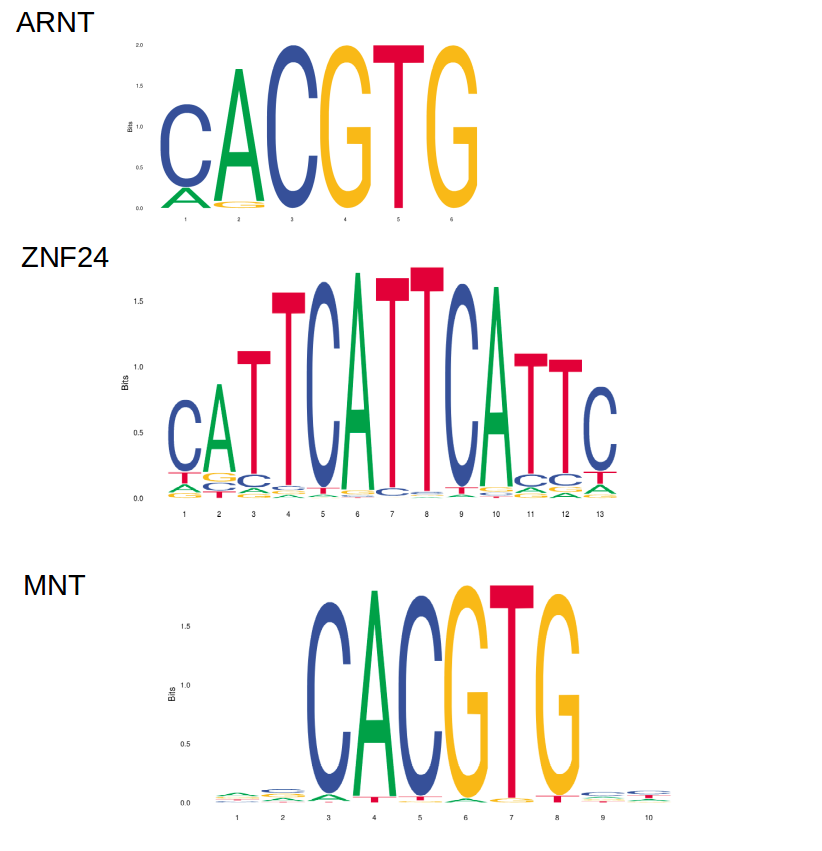


**Figure S3**

Pairwise comparisons of Euclidean distances between replicates of different proteins (inter) and within replicates of the same proteins (intra) in the original dataset and after removing the segments within the variable regions for the K562 ChIP-seq dataset (four proteins with three replicates).


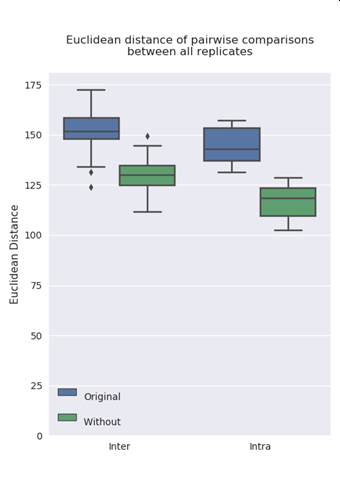

Supplement: gkaa180_Supplemental_Files [file gkaa180_supplemental_files.zip › Repro_Suppl_5.docx]
